# Office of the Children's Commissioner, London, United Kingdom

**DOI:** 10.23889/ijpds.v8i2.2209

**Published:** 2023-09-14

**Authors:** Sarah Taylor, Stephanie Friend

**Affiliations:** 1 Office of the Children's Commissioner, London, United Kingdom

The Children’s Commissioner, Dame Rachel de Souza, has a statutory remit to investigate the experiences of children in England. It is her duty to promote and protect the rights of all children, with particular regard to children who are living away from home or receiving social care services.

In doing this she regularly exercises her power to require government departments and other public bodies to provide administrative data, most of it at child level. The Commissioner’s research team aim to use cutting-edge methods and approaches to analysis of this data, and to triangulate findings with qualitative evidence from visits to settings such as schools, and the experiences of children the office represents directly.

This presentation will describe some of the office’s most sensitive and innovative recent and current research. These involve the gathering, linking and analysis of admin data in youth justice, education and children’s social care. Examples include novel analysis published 2022 of 512 Education Health and Care Plans; text analysis of over 13 million words of police interview transcripts conducted with children, published 2023; and novel linkage of education and social care data, published 2023.

The policy context and impact of the studies will be discussed, as well as methodological challenges and opportunities for future research by the office and in partnership with other researchers.

